# Effects of Acute Yohimbine Hydrochloride Supplementation on Repeated Supramaximal Sprint Performance

**DOI:** 10.3390/ijerph19031316

**Published:** 2022-01-25

**Authors:** Megan E. Barnes, Camryn R. Cowan, Lauren E. Boag, Julianne G. Hill, Morgan L. Jones, Kylie M. Nixon, Mckenzie G. Parker, Shelby K. Parker, Mary V. Raymond, Lillie H. Sternenberg, Shelby L. Tidwell, Taylor M. Yount, Tyler D. Williams, Rebecca R. Rogers, Christopher G. Ballmann

**Affiliations:** Department of Kinesiology, Samford University, 800 Lakeshore Dr. Birmingham, Homewood, AL 35229, USA; mbarnes4@samford.edu (M.E.B.); ccowan1@samford.edu (C.R.C.); lboag@samford.edu (L.E.B.); jhill18@samford.edu (J.G.H.); mjones22@samford.edu (M.L.J.); knixon@samford.edu (K.M.N.); mparke10@samford.edu (M.G.P.); sparker6@samford.edu (S.K.P.); mraymond@samford.edu (M.V.R.); lsternen@samford.edu (L.H.S.); stidwel2@samford.edu (S.L.T.); tyount@samford.edu (T.M.Y.); twilli11@samford.edu (T.D.W.); rrogers1@samford.edu (R.R.R.)

**Keywords:** power output, Wingate, lactate, epinephrine, norepinephrine

## Abstract

The purpose of this study was to examine the effects of a single acute dose of yohimbine hydrochloride on repeated anaerobic sprint ability. Physically active females (*n* = 18) completed two separate repeated supramaximal sprint trials each with a different single-dose treatment: placebo (PL; gluten-free corn starch) or yohimbine hydrochloride (YHM; 2.5 mg). For each trial, participants consumed their respective treatment 20 min before exercise. Following a warm-up, participants completed 3 × 15 s Wingate anaerobic tests (WAnTs) separated by 2 min of active recovery. A capillary blood sample was obtained pre- and immediately post-exercise to measure blood concentrations of lactate (LA), epinephrine (EPI), and norepinephrine (NE). Heart rate (HR) and rate of perceived exertion (RPE) were measured following each WAnT. Findings showed that mean power (*p* < 0.001; η^2^ = 0.024), total work (*p* < 0.001; η^2^ = 0.061), and HR (*p* < 0.001; η^2^ = 0.046), were significantly higher with YHM supplementation versus PL. Fatigue index (*p* < 0.001; η^2^ = 0.054) and post-exercise LA (*p* < 0.001; d = 1.26) were significantly lower with YHM compared to PL. YHM resulted in significantly higher EPI concentrations versus PL (*p* < 0.001; η^2^ = 0.225) pre- and post-exercise while NE only increased as a function of time (*p* < 0.001; η^2^ = 0.227) and was unaffected by treatment. While RPE increased after each WAnT, no differences between treatments were observed (*p* = 0.539; η^2^ < 0.001). Together, these results suggest that acute YHM ingestion imparts ergogenic benefits which may be mediated by lower blood LA and fatigue concomitantly occurring with blood EPI increases. Thus, YHM may improve sprint performance although more mechanistic study is warranted to accentuate underlying processes mediating performance enhancement.

## 1. Introduction

Yohimbine (YHM) is an indole alkaloid derived from the bark of the *Pausinystalia johimbe* tree, which is native to varying African regions [1]. Traditionally, YHM has been consumed through dietary enrichment with a ground extract of the native tree bark to improve feelings of energy and virility [2]. Due to inconsistencies in yohimbine concentrations in whole bark extract, yohimbine hydrochloride is more commonly used as a standardized form that is commercially available in stimulant and lipolytic supplements [3]. YHM ingestion induces a hyperadrenergic state which has been shown to induce losses in fat mass [4], increase blood flow [5], and alter fuel utilization [6]. Because of this, recreational and competitive athletes anecdotally use YHM alone or in combination with other supplements in an attempt to improve various facets of exercise performance. However, little to no research has investigated the efficacy of the potential ergogenic effects of YHM, leaving a great need for further research.

When consumed orally, YHM is rapidly absorbed (absorption half time: ~10 min) and is also quickly eliminated (elimination half-life: ~30 min) [7]. Thus, peak YHM levels following ingestion appear to occur between 20 and 30 min and can be fully eliminated within 60 min post-ingestion. While precise physiological mechanisms mediating YHM effects have yet to be fully elucidated, the majority of evidence demonstrates YHM possesses sympathomimetic properties [1,2]. The induction of sympathetic responses with YHM is underpinned by its antagonism of presynaptic α_2_-adrenergic receptors which typically results in elevations of blood norepinephrine (NE) [1,8]. Indeed, previous work has shown that infusion of YHM in healthy men results in increased blood epinephrine (EPI) and NE [9]. Bolstering this further, previous investigations have shown that YHM increases heart rate [10,11], systolic blood pressure [10,12], alertness [13], and localized blood flow [5]. While much of the literature regarding YHM and blood flow alterations exist in regard to treating impotence [14], it has been shown to induce constriction of splanchnic vessels, leading to shunting of blood and skeletal muscle hyperemia [7]. Increases in localized skeletal muscle blood flow may, in turn, enhance anaerobic exercise performance by controlling immediate energy system metabolism and LA production/removal [15,16]. However, whether YHM influences exercise performance has remained relatively unstudied, necessitating further investigation.

Despite the lack of research, YHM is a popular commercial supplement in the fitness industry and is widely distributed in the United States proverbially as a potent stimulant [3]. It can cause temporary adverse effects including tachycardia, hypertension, and flushing associated with sympathetic stimulation. However, reported cases of serious adverse events in exercise and sport have almost exclusively been observed with supraphysiological doses (5+ grams/dose) [17,18]. Literature on how YHM influences exercise performance is limited and inconsistent. Al-Kuraishy et al. showed acute YHM ingestion improved oxygen uptake kinetics and aerobic cycling performance in healthy males, which the authors postulated may be reflective of increased skeletal muscle blood flow [19]. Countering these findings, Stahlnecker et al. showed that a multi-ingredient supplement containing YHM failed to improve sprint performance despite increasing glycolytic flux and blood lactate concentrations [20]. However, the exact contribution of YHM to these results is unknown due to the amalgam of ingredients in the supplement. Previous evidence in male soccer players has also observed that chronic YHM supplementation did not improve bench press or vertical jump performance despite lowering fat mass at the end of the enrichment period [4]. Findings and disparities thereof are likely multi-faceted, occurring through inadequate blinding, varying training statuses of participants between studies, and heterogenous YHM dosing strategies [21]. Therefore, additional double-blinded placebo-controlled studies are direly needed before YHM can be attributed to possible performance enhancement.

While widely consumed in dietary energy supplements by recreational exercisers and athletes, little evidence to date supports a clear consensus as to the efficacy of YHM. Of the small amount of literature available, acute dosing may be most effective in performance enhancement [19]. Furthermore, previous reports of hyperlactatemia following YHM ingestion suggests that it may alter anaerobic exercise performance and fatigue over repeated sprints [20]. Therefore, the purpose of this study was to investigate the effects of an acute dose of YHM on performance during repeated cycle sprints. Furthermore, physiological indicators of the sympathetic response (i.e., catecholamines) and glycolytic metabolism (i.e., lactate) were measured. We hypothesized that YHM would increase sprint ability due to neural stimulation but also induce earlier decreases in power/increase fatigue over repeated bouts. Furthermore, we hypothesized that increases in catecholamines (epinephrine, norepinephrine) and blood lactate would be exacerbated by YHM supplementation.

## 2. Materials and Methods

### 2.1. Study Design

The present study utilized a double-blinded, counterbalanced, crossover design to examine the effects of acute yohimbine HCl (YHM) supplementation on repeated supramaximal sprint ability and associated responses of blood lactate (La), epinephrine (EPI), and norepinephrine (NE). Physically active females agreed to participate and completed two sprint trials, each with a varying treatment: (1) YHM and (2) placebo (PL; gluten-free cornstarch). Following supplementation, participants completed 3 × 15 s Wingate Anaerobic Tests (WAnTs). Blood was collected pre- and post-exercise. Each trial was separated by a 5 day washout period.

### 2.2. Participants

To determine the appropriate sample size, an a priori power analysis was conducted using G*power software (G*power V 3.1.9.4). A previous study from our lab that used an identical exercise protocol with a sympathomimetic supplement showed improvements in anaerobic capacity with supplementation with an effect size of f = 0.43 [22]. Accordingly, the minimum sample size was determined using the following parameters: test = repeated-measures ANOVA, α = 0.05, 1 − *β* = 0.8, *r* = 0.50. This was calculated to a minimum sample size of *n* = 12 for adequate power. Thus, eighteen physically active females (*n* = 18) participated and descriptive characteristics are displayed in (Table 1). To be deemed physically active, participants had to engage in ≥150 min/week of moderate-intensity exercise [23]. Exclusion criteria included the presence of lower-body injuries within 6 months before the initial testing date, chronic disease (cardiovascular, metabolic, or renal), and experience supplementing with yohimbine. Prior to each visit, participants were asked to refrain from vigorous lower-body exercise at least 24 h before testing [24]. Additionally, participants were asked to refrain from consuming caffeine, nicotine, and alcohol at least 12 h before testing [25]. Participants were encouraged to keep normal dietary and sleep habits prior to each trial. Preceding any data collection, verbal and written informed consent were obtained from each participant. All experimental procedures were conducted in accordance with the Declaration of Helsinki and approved by the Samford University Institutional Review Board.

### 2.3. Supplementation

Participants ingested either PL (gluten-free cornstarch) or YHM (2.5 mg; Primaforce, Burlington, NC, USA) 20 min prior to each trial. Contents were administered orally in an indistinguishable gelatin capsule. All treatments were distributed in a double-blinded manner, whereby an independent researcher organized non-identifiable opaque bags containing each treatment. To ensure compliance, empty bags were returned by participants and recorded. Color and shape of capsules between PL and YHM treatment were identical. Participants were not aware of any experimental hypotheses.

### 2.4. Blood Collection and Analysis (Lactate, Epinephrine, and Norepinephrine)

Approximately 500–600 μL of capillary blood was collected pre- and post-exercise through a finger prick, as previously described by our lab [26]. Briefly, a 17 gauge 2.0 mm depth disposable lancet was used to produce bleeding on the distal end of either the third or fourth finger. Initial drops of blood were used to measure blood lactate using a portable lactate meter (Lactate Plus Meter, Nova Biomedical, Waltham, MA, USA). The participant’s finger was massaged to promote bleeding and the remaining volume was collected by using capillary action into lithium-heparin coated Microvette^®^ tubes (SARSTEDT, Newton, NC, USA). Whole blood was then centrifuged at ~10,000× *g* rpm for 10 min. The separated plasma was decanted and subsequently stored at −80 °C until biochemical analysis after data acquisition. Plasma concentrations of EPI and NE were determined using a commercially available enzyme-linked immunosorbent assay (ELISA) kit (ABNOVA, Taipei, Taiwan) [26,27,28]. All samples were analyzed in duplicate and according to the manufacturer’s instructions and as previously described in our lab [26].

### 2.5. Procedures

For each trial, participants were equipped with a chest-strap heart rate monitor (Polar, Beth Page, NY, USA). A capillary blood sample was then obtained prior to exercise (pre) as aforementioned. Participants then completed a 5 min standardized warm-up at 50 watts on a mechanically braked cycle ergometer (Monark, Varberg, Sweden) to a metronome set to 60 bpm. For the repeated supramaximal tests, participants completed 3 × 15 s Wingate Anaerobic Tests (WAnTs), as previously described by our lab [22,24,29]. Participants performed the WAnTs on an electronically braked cycle ergometer (Velotron, Racermate Inc., Seattle, WA, USA). Seat height was standardized between visits for each participant. To begin each WAnT, participants pedaled for 20 s against an unloaded resistance that was immediately ensued by a 10 s lead-in phase to allow for the attainment of maximal pedal rate. At the end of the lead-in phase, resistance was immediately applied at 7.5% of the participant’s body mass and they pedaled as hard and as fast as possible for 15 s. Participants then completed 2 additional WAnTs for a total of 3 supramaximal tests with a separation of 2 min of active recovery. After each WAnT, RPE (1–10 scale) was documented. All participants were verbally encouraged during the testing. Then, identical blood collection protocols were repeated.

### 2.6. Data Analysis

Data analysis was completed using Jamovi software (Version 0.9; Sydney, Australia). Confirmation of data normality was conducted using the Shapiro–Wilk methods. A 2 × 3 [treatment × test] repeated-measures ANOVA was used to analyze all performance data, HR, and RPE. Test-to-test analysis and average performance (represents the main effect for condition) are shown. Furthermore, a 2 × 2 [treatment × time] was utilized to analyze blood La, EPI, and NE. A Bonferroni–Holm post hoc analysis was conducted for significant main effects or interactions. Estimates of effect size for main effects were calculated using eta squared (η^2^) and interpreted as: 0.01—small; 0.06—medium; ≥0.14—large [30,31]. Effect sizes between variable means were calculated via Cohen’s d (d) and interpreted as: 0.2—small; 0.5—moderate; 0.8—large [30,31]. Significance was set at *p* ≤ 0.05 a priori.

## 3. Results

### 3.1. Mean Power, Peak Power, Total Work, and Fatigue Index

**Figure 1:**
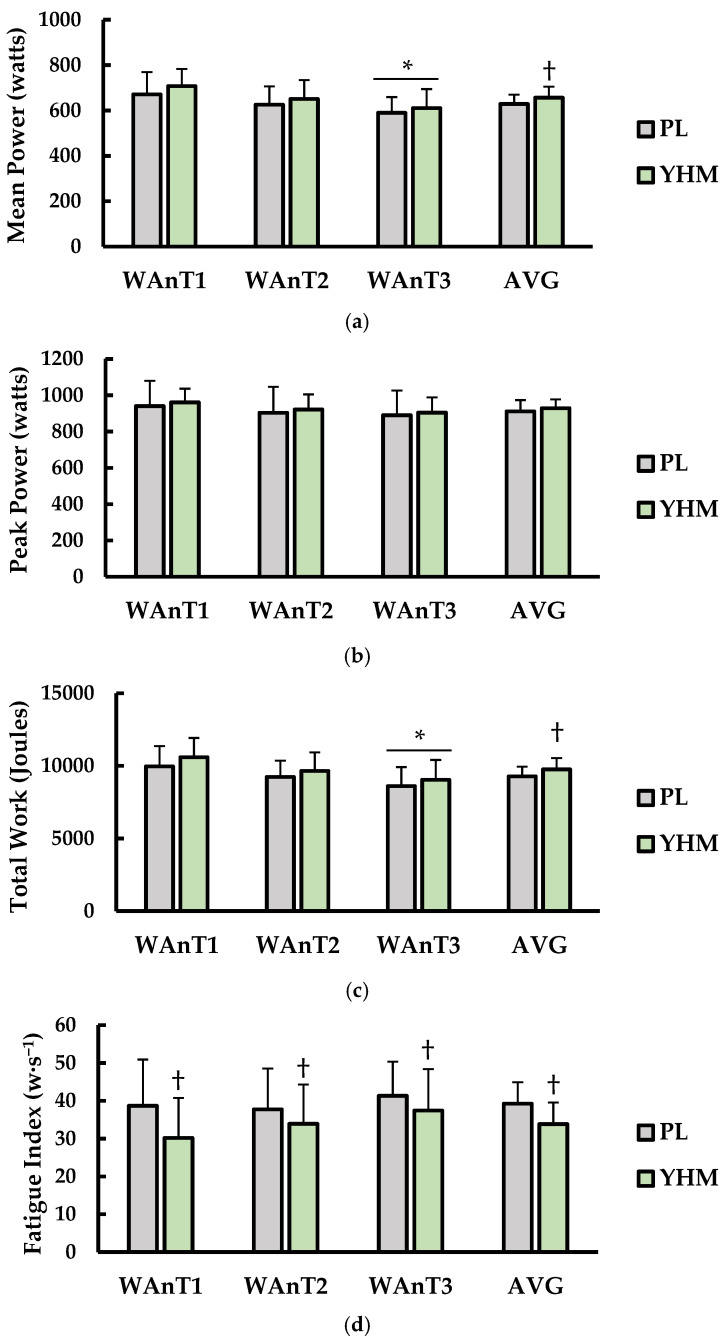
(**a**) Mean power (watts), (**b**) peak power (watts), (**c**) total work (joules), and (**d**) fatigue index (watts/s) compared between placebo (PL; grey bars) and yohimbine (YHM; green bars). Data are shown as the mean ± SD. Measurements are shown for WAnT1, WAnT2, WAnT3, and the average of all three tests together (AVG) for each condition. * indicates significantly different from WAnT1 (*p* < 0.05). † indicates significantly different from PL (*p* < 0.05).

Test-to-test and average performance (AVG) for mean power, peak power, total work, and fatigue index are shown in (Figure 1). For mean power (watts; Figure 1a), there was a main effect for treatment (*p* < 0.001; η^2^ = 0.024) and test (*p* = 0.001; η^2^ = 0.168) but not interaction between treatment × test (*p* = 0.528; η^2^ = 0.001). Specifically, YHM supplementation results in significantly higher AVG mean power compared to PL (*p* < 0.001; d = 0.61). Mean power for WAnT1 was significantly higher than WAnT3 irrespective of treatment (*p* = 0.004; d = 1.16). Peaker power (watts; Figure 1b) did not show a significant main effect for treatment (*p* = 0.058; η^2^ = 0.004) or test (*p* = 0.464; η^2^ = 0.026).

Furthermore, there was no interaction for treatment × test (*p* = 0.966; η^2^ < 0.001). For total work (joules; Figure 1c), there was a significant main effect for treatment (*p* < 0.001; η^2^ = 0.030) and test (*p* = 0.005; η^2^ = 0.030). No interaction of treatment × time (*p* < 0.001; η^2^ = 0.030) for total work was observed. YHM ingestion resulted in significantly higher AVG total work versus PL (*p* < 0.001; d= 0.64). Total work was higher in WAnT3 compared to WAnT1 regardless of condition. Fatigue index (watts·s^−1^), which represents the (peak power-minimum power)/duration of test, is presented in (Figure 1d). There was a significant main effect for treatment (*p* < 0.001; η^2^ = 0.054) but not test (*p* = 0.394; η^2^ = 0.032). There was an interaction between treatment × test (*p* = 0.007; η^2^ = 0.009). YHM ingestion resulted in a lower AVG fatigue index versus PL (*p* < 0.001; 1.08). Furthermore, YHM ingestion resulted in a lower fatigue index during WAnT1 (*p* < 0.001; η^2^ = 0.075), WAnT2 (*p* = 0.025; d = 0.36), and WAnT3 (*p* = 0.020; d = 0.39) compared to PL.

### 3.2. Heart Rate (HR) and Rate of Perceived Exertion (RPE)

**Figure 2:**
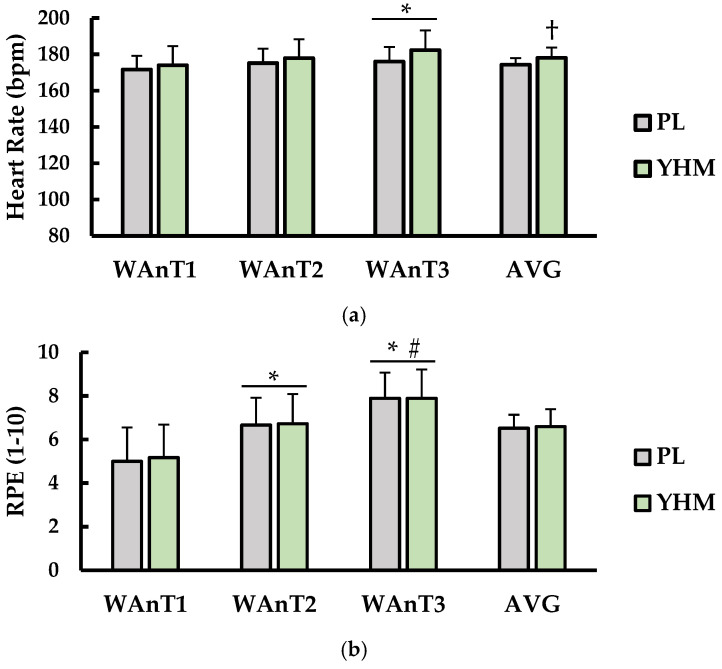
(**a**) Heart rate (HR; bpm) and (**b**) rate of perceived exertion (RPE; 1–10) compared between placebo (PL; grey bars) and yohimbine (YHM; green bars). Data are shown as the mean ± SD. Measurements are shown for WAnT1, WAnT2, WAnT3, and the average of all three tests together (AVG) for each condition. * indicates significantly different from WAnT1 (*p* < 0.05). # indicates significantly different from WAnT2 (*p* < 0.05). † indicates significantly different from PL (*p* < 0.05).

Test-to-test and average (AVG) values for heart rate (HR) and rate of perceived exertion (RPE) are shown in (Figure 2). For HR (bpm; Figure 2a), there was a main effect for treatment (*p* < 0.001; η^2^ = 0.046) and test (*p* = 0.045; η^2^ = 0.090). However, there was no interaction between treatment × test (*p* = 0.213; η^2^ = 0.010). AVG HR was significantly higher with YHM ingestion versus PL (*p* < 0.001; d = 0.97). HR during WAnT1 was significantly lower than WAnT3 regardless of treatment (*p* < 0.001; d = 0.82).

For RPE (1–10 scale; Figure 2b), there was a main effect for test (*p* < 0.001; η^2^ = 0.394) but not treatment (*p* = 0.539; η^2^< 0.001). There was also no interaction between treatment × test (*p*= 0.846; η^2^ < 0.001). Post hoc analysis showed that RPE during WAnT2 (*p* = 0.002; d = 1.08) and WAnT3 (*p* < 0.001; η^2^ = 0.394) was significantly higher than WAnT1. Furthermore, RPE during WAnT3 was significantly higher than WAnT2 (*p* = 0.013; d = 1.98).

### 3.3. Blood Lactate (LA), Norepinephrine (NE), and Epinephrine (EPI)

**Figure 3:**
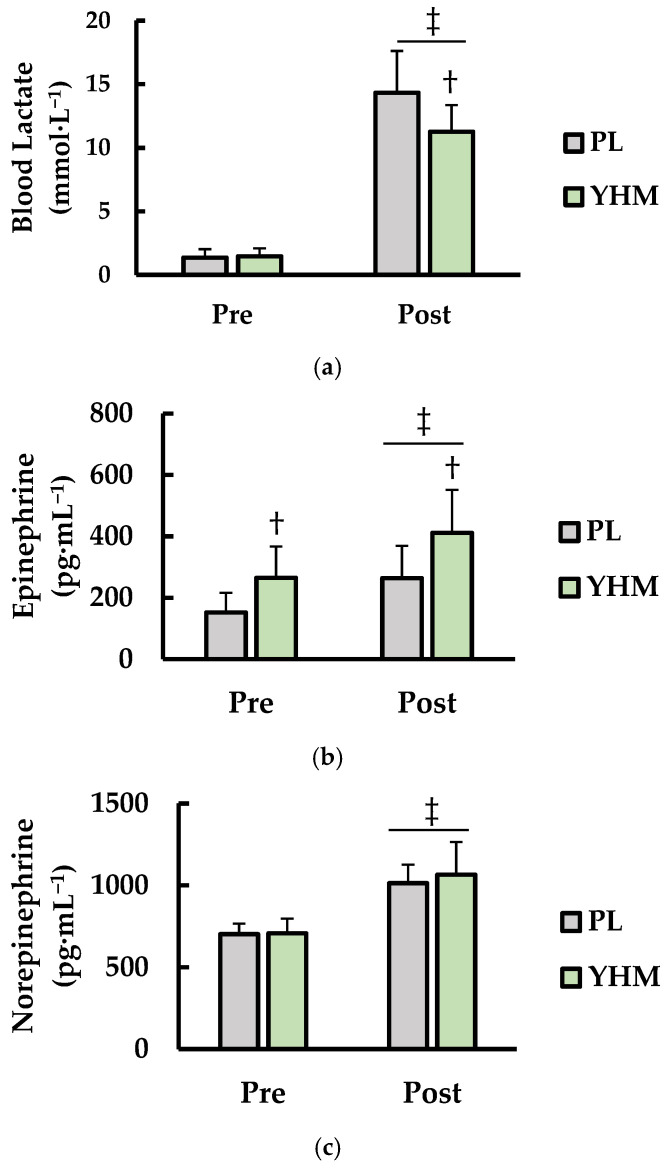
(**a**) Blood lactate (LA; mmol·L^−1^), (**b**) plasma epinephrine (EPI; pg·mL^−1^), and (**c**) plasma norepinephrine (NE; pg·mL^−1^), compared between placebo (PL; grey bars) and yohimbine HCL (YHM; green bars). Measurements were also taken and compared immediately prior to (pre-) and after (post-) exercise. † indicates significantly different from PL (*p* < 0.05). ‡ indicates significantly different from pre-exercise (*p* < 0.05).

Analysis of concentrations of blood LA (mmol·L^−1^), norepinephrine (pg·mL^−1^), and epinephrine (pg·mL^−1^) is shown in (Figure 3). For blood LA (Figure 3a), there was a main effect for treatment (*p* = 0.003; η^2^ = 0.015) and time (*p* < 0.001; η^2^ = 0.868). A significant interaction for treatment × time (*p* < 0.001; η^2^ = 0.017) was also observed. More specifically, LA was higher post- compared to pre-exercise (*p* < 0.001) regardless of treatment, and higher with YHM supplementation compared to PL (*p* = 0.047). However, YHM ingestion resulted in lower LA levels post-exercise compared to PL (*p* = 0.002). Analysis of EPI (Figure 3b) showed a significant main effect for time (*p* < 0.001; η^2^ = 0.225) and treatment (*p* < 0.001; η^2^ = 0.227). Furthermore, there was no interaction for treatment × time (*p* = 0.178; η^2^ = 0.004). EPI was significantly higher at post- compared to pre-exercise (*p* < 0.001). EPI was significantly higher from pre- to post-exercise regardless of treatment (*p* < 0.001). EPI was significantly higher with YHM treatment at pre- (*p* = 0.017) and post-exercise (*p* < 0.001). For NE (Figure 3c), there was a main effect for time (*p* < 0.018; η^2^ = 0.654) but not for treatment (*p* = 0.433; η^2^ = 0.005). No interaction for treatment × time (*p* = 0.429; η^2^ = 0.003) was observed. NE was significantly higher post- when compared pre-exercise (*p* < 0.001) irrespective of treatment.

## 4. Discussion

Despite limited research on efficacy, YHM is widely consumed for its stimulant properties and purported performance-enhancing effects [3,21]. To date, evidence of ergogenic effects is conflicting but suggests that acute dosing protocols may be most effective in inducing performance improvements [19]. Accordingly, this study sought to determine if acute YHM supplementation influences repeated anaerobic sprint performance and physiological responses to high-intensity sprint exercise. Our findings show that an acute dose of YHM ingested 20 min before exercise results in increased average power output and total work during repeated sprints. Although peak power was unaffected by treatment, fatigue index was markedly lower across all WAnTs with YHM supplementation. Of particular note, performance improvements contemporaneously occurred with increases in EPI and lower LA while NE was largely unaffected by treatment. Average HR was higher with YHM treatment without treatment-induced changes in RPE. Precise mechanisms for current findings are not fully clear, but these results support YHM as a stimulant capable of sympathoadrenal modulation and induction of greater work capacity.

Presently, an acute dose of YHM resulted in greater average mean power and total work compared to PL. This supports previous reports using a similar acute dosage albeit in endurance-based exercise. Al-Kuraishy et al. found that a single dose of YHM 2 h prior to exercise resulted in improved distance, time, and velocity of oxygen uptake maximum (vVO_2max_) during a standardized endurance cycling test [19]. While not physiologically confirmed in the study, the authors postulated that redistribution of blood flow from viscera to skeletal muscle may have resulted in performance improvements. Indeed, alterations in blood flow have been well documented with YHM through antagonism of α_2_-adrenergic receptors [5]. Pertaining to exercise performance, YHM may intensify splanchnic vessel vasoconstriction, ultimately leading to a greater fraction of cardiac output being directed towards active skeletal muscle. While speculative, current improvements in power and work output over the repeated sprints with YHM may be produced by increased blood flow to active lower limb muscle mass. Previous evidence has shown that phosphocreatine (ATP-PC) resynthesis is highly dependent on oxidative ATP production by which increases in blood flow to the muscle may accelerate ATP-PC recovery [32]. Thus, it is plausible that YHM may have improved blood flow to the lower limbs, thereby increasing ATP-PC resynthesis rates and immediate energy availability. However, other conflicting evidence has shown that YHM does not benefit anaerobic power. For example, Ostojic et al. found that a 21 day YHM regimen did not improve shuttle run time or vertical jump height in elite male soccer players [4]. Disparities in findings are not fully understood but may be related to the YHM supplementation period. Given that Ostojic et al. gave participants YHM for a total of 21 days, there may have been desensitization to the effects of YHM over time. Bolstering this, many other stimulants have been reported to lose potency, whereby cellular responses to treatment are blunted and treatment effects are diminished in a transient dose-dependent manner [33,34]. Therefore, our results support the use of acute dosing with YHM for improved anaerobic performance, and future research is needed to determine the optimum dose, supplementation duration, and ingestion frequency to optimize performance further.

Counter to our hypothesis, metrics of fatigue were improved or unchanged with YHM ingestion. Fatigue index, which indicates power output loss over time, was significantly lower with YHM, indicating that supplementation at least partially allowed participants to better maintain power output throughout the repeated sprints. The lack of published mechanistic research on YHM does not allow the current study design to fully rationalize fatigue improvements. However, α_2_-adrenergic agonists, which induce the opposite action of YHM, have been shown to induce fatigue and decrease alertness [35]. Supporting fatigue attenuation, LA was also significantly lower post-exercise with YHM, suggesting possible improvements in LA clearance or lower LA production. Collectively, lower fatigue with YHM ingestion may be manifested in sympathoadrenal-mediated hemodynamic change as well. As previously mentioned, redistribution of blood flow to skeletal muscle may aid in ATP-PC resynthesis, improving immediate ATP production [32]. However, it might also synergistically aid in LA and metabolite production/removal. While theoretical, possible increases in ATP-PC recovery with YHM may partially attenuate the need for accelerated glycolysis to meet ATP demand, thus resulting in lower LA production. Veritably, previous work has shown that enhancement of ATP-PC stores decreases blood LA during intense exercise, which was most conspicuous in type II muscle fibers [36]. Furthermore, YHM-mediated redistribution of blood flow may improve the removal of lactate/H^+^, thereby attenuating pH-associated muscular fatigue. YHM has been shown to alter regional hemodynamics, namely decreasing blood flow to intestines and kidneys via changes in α-adrenergic receptor activity [37]. Changes in blood flow redistribution to the muscle under α-adrenergic receptor control appear to be most pronounced in the contracting skeletal. Rauwolscine, a YHM analog and potent α_2_-adrenergic receptor antagonist, has been shown to increase vascular conductance and blood flow to active skeletal muscle by ~20%, which may also reflect current changes [38]. Increases in EPI, similar to that seen in the current investigation, have also been shown to stimulate the metabolic rate of non-exercising skeletal muscle, thereby conceivably favoring LA transport and oxidation albeit in rodent muscle, not human [39]. However, the reader is cautioned as blood flow and LA handling were not directly measured, pointing to the need for more mechanistic studies of how YHM may alter local control of skeletal muscle blood flow during intense exercise and how this may alter arterio-venous LA.

YHM has been repeatedly shown to increase catecholamine levels, principally NE [1,11,40]. In the current study, acute YHM ingestion resulted in greater EPI levels pre- and post-exercise while NE increased post-exercise independent of treatment. With the study design currently utilized, the lack of change in NE with YHM treatment is difficult to explicate as this is in direct opposition to previous work. Lack of agreement with previous findings may be due to biological sex differences in response to supplementation. To date, we are unaware of any studies which have studied YHM ingestion and exercise in females, further supporting the widespread need for studies investigating female responses to exercise [41,42]. Females have been shown to exhibit less pronounced sympathetic responses and lower circulating blood NE levels compared to male counterparts [43]. Indeed, Gratas-Delamarche et al. showed that females had lower catecholamine and LA levels compared to males following a single 30 s WAnT [44]. Females have also been shown to exhibit less NE spillover during exercise compared to males [45,46]. Thus, females may have extenuated NE responses compared to males which may have dampened NE spillover with YHM treatment. Interestingly, YHM incited marked increases in EPI pre- and post-exercise. Although less commonly reported, YHM has been shown to result in elevated blood EPI levels in humans [10]. Reasons for performance enhancements from YHM-mediated increases in EPI are likely multi-faceted. First, the increase in EPI pre-exercise may have resulted in the aforementioned blunted LA accumulation. Trudeau et al. showed that pre-exercise treatment with exogenous EPI infusion attenuated blood LA increases following exercise [47]. The authors postulated that re-release of EPI during exercise following the pre-exercise infusion may exacerbate visceral vasoconstriction further, thereby accentuating blood flow redistribution to muscle. This would ultimately influence LA clearance and majorly supports current findings of lower fatigue and blood LA concentration [47]. During exercise, the increased EPI levels may have systemically increased blood flow and initiated inotropic effects. EPI may alter cardiac function directly by increasing heart rate, which was currently observed, and through increased cardiac contractility. This would lead to greater cardiac output and blood circulation supporting blood flow alterations further. Indeed, these alterations may lead to higher venous return thus affecting cardiac contractility [48]. The rationale for this is well supported as YHM has been shown to initiate catecholamine alterations in NE release and improve cardiac contractility [49,50]. Furthermore, high blood EPI during exercise with YHM ingestion may have positively influenced muscle contraction. EPI release alters neuromuscular processes during contraction both directly and indirectly [51]. For example, induction of sympathetic stimulation with YHM may have altered fiber type recruitment and muscle relaxation rates. Roatta et al. previously showed that physiological sympathetic stimulation increased motor unit firing rates, muscle twitch amplitude, and hastened relaxation time [52]. YHM-mediated EPI release may therefore impart ergogenic benefits through enhancement of both hemodynamic and inotropic mechanisms and further research is needed to determine precise contributions of each to resulting performance.

While the current study is the first to investigate acute YHM ingestion and repeated sprint performance, there were weaknesses that should be highlighted. First, much of the benefits with YHM are likely mediated through changes in blood flow [1,2,19]. Admittedly, this study lacked the ability to discover detailed mechanistic insight into blood flow with YHM, leaving most of the discussion to this point speculative. Much more study is needed in this area and reasons for performance enhancement will remain elusive until completed. Additionally, the menstrual cycle of participants was not strictly controlled for, which could possibly influence results. However, it is worth noting that our rationale for omitting this control is due to previous evidence showing that anaerobic performance is unchanged regardless of menstrual cycle phase in addition to the fact females are grossly understudied in exercise research [42,53,54]. The physiology of YHM supplementation in females is an area of dire need especially in the context of male comparison. Lastly, optimal dosing strategies for performance enhancement with YHM were not fully resolved. We cannot predict whether current effects will translate to higher/lower doses or how chronic ingestion influences efficacy. Thus, future investigations should implement direct comparison for varying dosages and regimens.

## 5. Conclusions

In conclusion, acute YHM ingestion increases both the ability to generate and maintain power output during repeated sprints. Likely, these improvements were underpinned by increases in EPI and lower blood LA, despite the lack of treatment-mediated NE increases. However, many proposed mechanisms remain speculative, highlighting the need for more mechanistic work. From a practical standpoint, this study suggests that athletes and exercisers looking to attenuate fatigue and improve anaerobic performance may use an acute YHM dose for ergogenic effects. However, this study also highlights the need for further study for how female exercise responses may differ from male counterparts with YHM supplementation. Given the lack of research profundity in biological sex-related differences to YHM treatment, current recommendations for its use may heavily favor males and inaccurately portray YHM effects to the general population.

## Figures and Tables

**Table 1:** Descriptive characteristics of female participants (*n* = 18).

Characteristic	Mean ± SD
Age (yrs)	20.1 ± 0.5
Height (cm)	169.2 ± 6.3
BM (kg)	67.6 ± 9.1
BMI (kg·m^2^)	23.8 ± 7.6

BM (body mass); BMI (body mass index).

## Data Availability

All data are available within this manuscript.
